# Second Mesiobuccal Root Canal of Maxillary First Molars in a Brazilian Population in High-Resolution Cone-Beam Computed Tomography 

**DOI:** 10.22037/iej.v13i1.18007

**Published:** 2018

**Authors:** Claudia Rezende Gomes Alves, Márcia Martins Marques, Maria Stella Moreira, Sueli Patricia Harumi Miyagi de Cara, Carlos Eduardo Silveira Bueno, Cesar Ângelo Lascala

**Affiliations:** a *Department of Radiology, Dental School, University of Sao Paulo (USP), Sao Paulo, SP, Brazil; Claudia Rezende Gomes Alves; *; b * Department of Endodontics, Dental School, University of Sao Paulo (USP), Sao Paulo, SP, Brazil; *; c * Dental School, Ibirapuera University (UNIB), Sao Paulo, SP, Brazil; *; d * Department of Dentistry, Dental School, Faculdades Metropolitanas Unidas (FMU), Sao Paulo, SP, Brazil; *; e * Department of Endodontics, Dental School, University of the Sao Paulo State (UNESP), Araçatuba, SP, Brazil*

**Keywords:** Cone-beam Computed Tomography, High Resolution, Maxillary First Molar, Mesiobuccal Root, High Resolution

## Abstract

**Introduction:**

The second canal of the mesiobuccal root (MB2) of the maxillary first molars (MFM) is difficult to detect in conventional radiographs and can be a major cause of failure in endodontic treatments. The aim of this study was to investigate the prevalence and anatomy of the MB2 by using high-resolution cone-beam computed tomography (CBCT).

**Methods and Materials:**

Three radiologists examined 414 high-resolution CBCTs. Of these, the CBCTs of 287 patients (mean age 49.43±16.76) who had at least one MFM were selected, making a total of 362 teeth. Prevalence and its relation with gender and age of the patients, side of the tooth, and Vertucci’s classification were analyzed. Data were statistically analyzed (*P*<0.05).

**Results:**

A total of 68.23% of the teeth exhibited the MB2. The presence of the MB2 was equivalent in both genders and significantly higher in younger patients. There was no correlation between the presence of the MB2 in relation to both the sides of the MFM and the FOV size. Smaller FOV recognized higher Vertucci’s grades.

**Conclusions:**

It was concluded that the prevalence of the MB2 canal in maxillary first molars in this Brazilian population examined with high-resolution CBTCs is 68.23%, being more prevalent in young patients. Gender and the side examined are no factors for determining the presence of MB2. Although the both FOVs of the high-resolution CBTCs (FOV 8 and 5) detect the MB2 canal, smaller FOV (FOV 5) is more accurate in the analysis of the internal anatomy of such root canals, according to the Vertucci´s classification.

## Introduction

First molars may present a second canal at its mesiobuccal root (MB2), which is usually small with large anatomical variations [1]. These canals are difficult to recognize in conventional radiographs (two-dimensional) [2], and may be an important cause of failure of endodontic treatments [3]. 

Root canal morphologies can be analyzed by some commonly used methodologies including root canal staining [4], tooth clearing [5], and conventional and digital radiographs [6]; each of them presenting limitations that could be circumvented by the use of a more sensitive imaging method such as cone-beam computed tomography (CBCT) that has been used as an auxiliary exam in cases of endodontic treatment [7-9], to evaluate periapical lesions [10] as well as root resorption [11]. Further, it would be important to use higher resolution equipment, which potentially has the ability to reveal the anatomical details of the root canal more efficiently and clearly [7-9].

The improvements in the software of the equipment must also be highlighted. These have greatly contributed to the best viewing and consequent enrichment of relevant information and differential diagnosis of reports and interpretations, mainly to be viewed simultaneously in all multiplanar and 3D reconstructions.

On the basis of the above, CBCT was used in this retrospective study, where the prevalence and anatomy of the MB2 of the maxillary first molar was analyzed using a high-resolution CBCT scanner. The importance is justified not only to overcome the difficulty of viewing this canal in other radiographic techniques, but also because this is the first study that investigates the morphology (*i.e.* the internal anatomy) of this canal with high-resolution CBCT using Vertucci's classification [12]. Additionally, this study called attention to the importance in detecting the MB2 in order to prevent possible unsuccessful endodontic treatment.

## Materials and Methods

This retrospective study was approved by the School of Dentistry of the University of Sao Paulo Research Ethics Committee (#1.235.239/2015).

CBTC scans that were evaluated in this study were requested by professionals from different specialties of dentistry, such as, endodontists, surgeons, periodontists and implantologists, for different diagnostic purposes in the time period from 10/01/2015 to 22/12/2015. Four hundred and fourteen CBCTs of the jaws of small field of view (FOV) (5 or 8) and high-resolution taken during the two-month interval were evaluated retrospectively. These CBCTs were obtained from 265 female patients and 149 males.

For the study inclusion and exclusion criteria were used to select the CBTCs. Inclusion criteria were: presence of at least one maxillary first molar with root canals filled or not. Exclusion criteria were presence of artefacts generated by pins and/or metal restorations that could interfere with the evaluation of the dental element, creating doubt in the interpretation of the examiners. After using these criteria, CBTCs of 186 women and 101 men, aged between 9 and 93 years (mean 49.43±16.76), were selected totaling 362 teeth.

The images were acquired with a cone-beam computed tomography (CBCT) in high-resolution, in accordance with the principles of ALARA (As Low as Reasonably Achievable) in a Prexion 3D Elite model XP68 (PreXion Inc., San Mateo, California, USA), with high-resolution protocols, and viewed in 3D software PreXion Image Analysis System (PreXion Inc. San Mateo, California, USA). The parameters of the acquisition protocol are described in Table 1.

The images selected by the above inclusion/exclusion criteria were evaluated in the 3D software PreXion Image Analysis System (PreXion Inc.) in i5 427 1.8 GHz with 4 GB RAM memory 32 GB SSD HD and Dell UltraSharp U2412M 24-inch monitor with a resolution of 1920×1200 by three expert examiners in Oral Radiology and Oral Imaging. These examiners, with at least five years of experience in CBCT examinations, were previously trained and calibrated. All volumes acquired from the maxillary first molars (right side and/or left side) were analyzed by using PreXion 3D Image Analysis System software.

The examiners determined the presence or absence of the MB2 canal in axial sections. When present, the MB2 canals were classified according to the classification established by Vertucci [12] in axial, sagittal and coronal sections.

Data from the examiners were compiled and tabulated in Excel spreadsheet 2007 software (Microsoft Corp., Redmond, WA, USA). Three sheets were prepared with the tooth-side data (16 or 26), gender and age of each patient, presence or absence of the MB2, size of FOV (5 or 8) used to obtain the image, and the classification of the MB2 canal when present according to Vertucci’s classification [12], preserving the identity of the patients. The software used for statistical analysis was the BioEstat-5.3 (Mamirauá Institute, Belém, Pará state, Brazil).

To evaluate the reproducibility in the detection of the MB2 canal in 362 teeth, the inter observer Kappa test was used. The three examiners (CGA, SGA and MAOS, all oral radiologists), observed the presence or absence of the MB2 canal data, and two by two were compared. A high reproducibility inter observer was observed, as follows: Observers 1 and 2 (Kappa 0.88; *P*<0.0001), 1 and 3 observers (Kappa 0.79; *P*<0.0001), and observers 2 and 3 (Kappa 0.85; *P*<0.0001). 

We compared the data of the patients with the MB2 canal with those of patients who did not have this canal. The comparison of gender was done by the Kruskal Wallis test, whereas the comparison of the age of these patients was achieved by the Student´s *t* test. The Pearson’s correlation test was used to compare the presence and absence of the MB2 canal with the side examined, and also to the FOV used to obtain the images (FOV 5 or FOV 8). The Pearson’s correlation test was also used in the comparative analysis between the size of the FOV and the scores of Vertucci’s classification [12].

## Results

The CBCTs of 362 maxillary first molars were obtained from 287 patients (186 female and 101 male). Seventy-five of these patients had two maxillary first molars in CBCT examinations.

**Figure 1:**
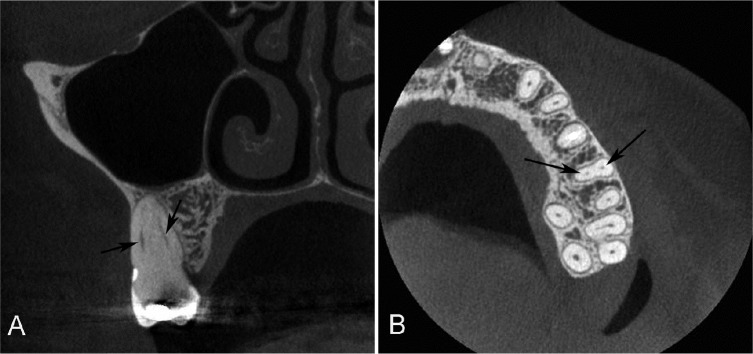
Cone-beam computerized tomography (CBCT) images of maxillary first molars with 2 canals (black arrows) in the mesiobuccal root as viewed in the coronal and axial directions. *A)* Coronal view with the MB root exhibiting type II canal configuration; *B)* Axial view

From the 362 teeth examined, 239 (66.02%) were from females and 123 (33.97%) from males. Out of these, 247 exhibited the MB2 canal giving a prevalence of 68.23%. There was no correlation between gender and the presence or absence of the MB2 (*P*=0.14; *r*=-0.076). Therefore, despite the number of examined teeth of female patients was higher than those of male patients, the prevalence of the MB2 canal was equivalent in both genders.

The age of the selected patients (287) for the study ranged from 9 to 93 years, with a mean of 49.43±16.76 years. The MB2 canal was detected in patients with significantly younger ages (mean age=45.04±18.386 years) than those of patients where the MB2 canal was not observed (mean age=53.46±14.995 years; *P*=0.0001).

There was no correlation between the side of the maxillary first molar and the prevalence of the MB2 canal (*P*=0.53; *r*=0.0327).

Out of 362 teeth, 190 were examined in CBTCs taken with FOV 5, and 172 teeth with FOV 8. There was no correlation between the sizes of the FOV used to obtain the images and the ability to detect the MB2 canal (*P*=0.09; *r*=0.087).

According to Vertucci’s classification [12] the MB2 canal of the maxillary first molars studied presented mainly the type I and II configurations (Figure1), and 22.38% of the roots were classified as type IV or higher. Thus, 22.38% of the roots not only presented a MB2 canal but also presented more than one apical foramen (Table 2). 

To verify if the size of the FOV would influence the determination of the Vertucci’s classification scores, two statistical analyses were done. Firstly, the Pearson’s correlation test between the FOV sizes and Vertucci's scores was done. There was a negative correlation (*P*=0.008; *r*=-0.140) between the FOV sizes and the Vertucci’s classification scores. In images obtained at a smaller FOV (FOV 5) the examiners observed higher Vertucci’s scores, while in the images of larger FOV (FOV 8), mostly smaller scores were observed. Secondly, the Vertucci’s classification scores of FOV 5 images were compared with those of FOV 8 by the Kruskal Wallis test, which confirmed the results of the correlation test (*P*=0.012) (*e.g.* the mean Vertucci’s classification scores were significantly higher in the imaging group of FOV 5 and smaller in those of FOV 8).

Out of the 287 patients evaluated, 75 (26.13%) presented two maxillary first molars. Of these, 44 (58.66%) showed the MB2 canal on both teeth, 17 (22.66%) had the MB2 canal in only one of the two molars, and 14 (18.66%) did not exhibit MB2 canal in any side. Thus, on 58 patients (77.33%) where both maxillary first molars were examined, the teeth of both sides presented the same characteristic in relation to the presence or absence of the MB2 canal. Moreover, patients with the MB2 canal in the teeth of both sides were significantly younger (33.95 years) than those who did not have the MB2 canal (49.35 years) on either side (50.57 years; *P*<0.05), or even those with the MB2 canal observed on only one side (*P*<0.05). There was no significant difference between the ages of patients with no MB2 canal in one or both sides.

## Discussion

The interpretation of the second canal of the mesiobuccal root (MB2) of the maxillary first molar in two-dimensional images is difficult due to its small diameter and large anatomical variation [13]. This can lead to endodontic treatment failure, particularly in root canals classified by Vertucci [12] as type IV or above (types V, VI and VII); where there are two isolate apical foramens, which may be not appropriately sealed at treatment ending. This could be circumvented by the use of CBCT not only in this clinical situation, but also for others where there is the presence of accessory canals. In fact, the American Association of Endodontics [14] highlights the importance of CBCT not only for the research and evaluation of root canals, but also for diagnosing fractures, internal and external root resorption, atresia, *etc.*

Currently, CBCT is widely used in dentistry, and therefore, there is the possibility of performing both prospective and retrospective clinical trials in the search for a variety of information. On the basis of this possibility, in the present study, a retrospective search was conducted in CBCTs obtained for dental diagnoses purposes in a Brazilian population. Examinations depicting at least one maxillary first molar in the CBTCs were selected. 

From 362 teeth examined, the observed prevalence and internal anatomy of the MB2 of the maxillary first molar was as high as 68.23%, and Vertucci´s type II [12] was the anatomy most commonly found in this root. Furthermore, the CBCTs were able to identify the anatomies of Vertucci´s type IV classification or above (types V, VI and VII) in 22.38% of roots, those with more than one apical foramen.

It is fundamental to note that these results were obtained only because a high-resolution scanner (Prexion Elite 3D) was used. This equipment features a small acquisition area (small FOV), reduced voxels, geometric projection, and collimation capacity. In fact, there are only a few reports of research on the MB2 carried out on equipment with these characteristics [15, 16].

The micro computed tomography (Micro-CT) of maxillary first molars show clearer details of the internal anatomical structure such as loop, accessory canals and intracanal communications with greater precision than any other method. This method is considered as the gold standard for understanding the complex root system [17]. However, this exam can only be performed *in vitro*, and therefore cannot be used by the endodontist, except at the research level. Still, in a comparative analysis between CBTC, digital periapical radiographs, and micro-tomography to check the number of canals in the mesiobuccal roots of maxillary molars, there were no statistically significant differences between the data of CBCT and micro tomography; but there were only between these and periapical radiographs were found [18]. Thus, these data should encourage endodontists to use CBCT as the imaginologic clinical exam of choice in their clinical routine.

On the basis of the inter observer analysis of findings, it was possible to show how the interpretation of the images obtained by this technique is easily reproducible once trained observers are able to consistently observe the presence or absence of the MB2 canal in the images. This high reproducibility in MB2 detection is consistent with the findings of other studies, which found no statistically significant differences in inter- and intra-observer assessments; or reported an accuracy of 98% in CBCT in detecting the MB2 [15, 18].

After determining the reproducibility of CBCT, the presence of the MB2 canal in the maxillary first molars was investigated, showing a high prevalence (68.23%) of this canal in the sample studied, which is similar to findings in other studies. Several authors have reported the prevalence of the MB2 canal in different populations using CBCT. Despite the variations related to sample size, age, and most of the methodologies used in these different surveys, the vast majority of authors reported that the prevalence of the MB2 canal when searched through CBCT is greater than 50% [13, 15, 17-21]. In fact, in a recent publication, Betancourt *et al.* [22] have found a very similar prevalence of the MB2 canal in another South American country (Chile) that was 69.82%.

The study of Plotino *et al.* [23] showed a low prevalence of the MB2 canal (40.3%); this result was probably based on an inadequate choice of the FOV size for the CBCT examinations which was 15 cm×15 cm, which rules out a high resolution image. Moreover, Silva *et al.* [1] found the MB2 in only 42.62% of teeth examined in a Brazilian population. It is noteworthy that this study was conducted with lower resolution equipment (i-CAT 3.1.62; Xoran Technologies, Ann Arbor, MI, USA) and even more focused on only the presence or absence of the MB2, and the internal morphology of these channels had not been studied. 

**Table 1:** Parameters of the acquisition protocol

**Parameters**		**C** **BTC**
**Work regimen**		90 Kvp and 4 mA
**Acquisition time**		37 s (high density mode)
**Sensor**		Flat panel with an area of 14 cm × 14 cm
**Acquisition Volume**		Cylindrical
**FOV size: **	**FOV 8**	8.1 cm × 7.5 cm (total jaw)
	**FOV 5**	5.6 cm × 5.2 cm (partial jaw)
**Rotation Degrees:**		360 degrees
**Gray scale**		14 bits
**Voxel size**	**FOV 8**	0.15 mm
	**FOV 5**	0.11 mm
**Focal point:**		0.15 mm
**Number of images**		1024

**Table 2:** Anatomy of the teeth in function with Vertucci´s classification

**Classification**	**Type I**	**Type II**	**Type III**	**Type IV**	**Type V**	**Type VI**	**Type VII**	**Type VIII**
**Number of teeth**	114	138	29	43	3	20	15	0
**Percentage of the total number of teeth **	31.49	38.12	8.01	11.87	0.82	5.52	4.14	0

As this is the first study of its kind conducted in a Brazilian population, this study analyzed not only the prevalence, but mainly the internal anatomy of the MB2 with high-resolution CBCT along with the characteristics of the patients, such as the gender and age. Despite of the number of examined teeth in women was higher than in men, there was no statistically significant difference in the prevalence of the MB2 canal in the first maxillary molars. In fact, other authors observed the same, namely that the presence of the MB2 had no correlation with gender [13, 21]. To now, we were able to find only in a study in a Korean population, a significantly higher prevalence of the MB2 canal in male patients was observed [20], but without an explanation for that finding.

The MB2 appeared more in younger patients. These results converge with those reported by Zheng *et al.* [15] and Kim *et al.* [20] who used high-resolution images. In contrast, other authors showed a greater prevalence of the MB2 in older patients [13, 19]. However, these studies showed significant biases related to samples or included many different ethnic groups; and used equipment with a FOV of 15cm×15cm, which is lower resolution [13] or studied populations with more elderly patients in their sample [19]. 

According to Iqbal and Fillmore [24], the age of the patients is an important preoperative predictor for the detection of extra canals in maxillary molars. A review of the literature on the extra root canals in the maxillary molars showed that they occur mostly in patients younger than 40 years old [25]. It may be assumed that with age, root canals or their orifices may calcify; making the detection of extra canals more difficult [24]. Thus, bearing in mind that during the tooth lifetime, there is a progressive mineral deposition in the light of its canals; so it would be expected that over time, even if the MB2 would be present, this could be completely obliterated in during the patient's life, especially because this canal has little light and then it will be no longer detected, even in high-resolution CBCT.

The side of the molar did not influence the prevalence of the MB2 canal, as observed by other authors [13, 15, 20, 23]. Only one study found a higher prevalence of the MB2 on molars on the left (91%) than on the right molars (81.6%), and the authors attributed this result to the sample studied, which had a higher number of molars on the left side [26]. In the present study, 75 patients (26.13%) presented maxillary first molars on both sides, showing that 58 patients (77.33%) had teeth both with the same characteristics of the presence or absence of the MB2 canal, and only 22.66% of the patients had teeth with different features, (*i.e.* the presence of the MB2 in only one side). Furthermore, it was found that when the two sides had the MB2, the teeth were from younger patients. Thus, the patient exhibiting the MB2 on only one side could have had the MB2 in both teeth when younger, and one of these canals could be obliterated when the time passed. These results could indicate the existence of an individual characteristic in the possibility of whether or not they have the MB2. This may be relevant for clinicians who, facing the detection of the MB2 canal in one of the maxillary first molars, could assume that the other molar also could have it, even if it was not detected in the imaginologic exam used.

In addition to determining the prevalence of the MB2 canal in maxillary first molars and its relationship to gender and age of patients, the internal anatomy of these canals was classified according to the classification proposed by Vertucci [12]. There were no roots identified as type VIII, *i.e.*, the mesiobuccal roots of the maxillary first molars of the examined population showed only one or two canals. As expected, the type II was the most found score (38.12%); however, in the CBTCs it was possible to observe anatomies of types IV or higher in 22.38% of the roots, which present more than one apical foramen. This result, in our view, was the most important finding, since the CBCT in the parameters used in this study were able to identify these roots as potential candidates for failure if they have to be subjected to endodontic treatment. 

In fact, conventional radiographs, even digital radiographs, barely detect the MB2 [18], so they would hardly show the MB2 internal anatomy finished at the dental apex as a single canal having its own foramen or foramina. In this case, the possibility of this canal being sealed adequately is not quite large. This finding strongly indicates that Endodontists can take advantage of relevance when using CBCT in their clinical routine [14]. Moreover, this CT scan should preferably use small FOV, since more detailed anatomy information was obtained when small FOV was used in the acquisition of the CBCT images. In fact, despite that FOV size was not a factor for the identification of the MB2, it was essential for the identification of different internal anatomies of the tooth roots analyzed in the CBCT images.

The present study showed that the prevalence of a second canal in the mesiobuccal root of maxillary first molars in the examined Brazilian population is high, and independent of gender or side of the tooth; but is more common in young patients and have a tendency, when present, to occur in both sides of the jaw. Moreover, there are roots with internal anatomies that indicate the existence of two apical foramens. These data are of great importance, and endodontists must be aware of it, since not only the fact that the second canal is of small volume, its anatomy can be responsible for the failure of endodontic treatment, and once when it is not obturated, leaves the apical foramen open and the canal exposed at the treatment ending. Therefore, professionals are encouraged to request high-resolution CBCT and low FOV before performing endodontic treatment in maxillary first molars, given that this examination is able to highlight this channel, and reveal its anatomy in detail. 

According to Skidmore and Bjorndal [27], genetics determines the internal complexities of the root canal. Therefore, the study also had its value in relation to the knowledge of the anatomy of the mesiobuccal root of maxillary first molars in another population, Brazilians, which is quite heterogeneous, formed by immigrants from countries of all continents of the world. This study adds to other studies in the literature that have examined other populations, such as North American [13], Chinese [28], Korean [20], Greek [21], Turkish [29], among others, providing data for future research of comparisons between ethnic characteristics in determining the presence of more than one canal in this root.

The prevalence of a second canal in the mesiobuccal root (MB2) of maxillary first molars is high. Professionals are encouraged to request high-resolution CBCT with low FOV, which would avoid endodontic treatment failure by revealing MB2 internal anatomy in detail.

## Conclusion

It was concluded that the prevalence of the MB2 canal in maxillary first molars in this Brazilian population examined with high-resolution CBTCs is 68.23%, being more prevalent in young patients. Gender and the side examined are no factors for determining the presence of MB2. Although the both FOVs of the high-resolution CBTCs (FOV 8 and 5) detect the MB2 canal, smaller FOV (FOV 5) is more accurate in the analysis of the internal anatomy of such root canals, according to the Vertucci´s classification.
